# The Address in Obstetrics

**Published:** 1902-08-02

**Authors:** William Japp Sinclair

**Affiliations:** Professor of Obstetrics in the Owens College, Manchester.


### THE ADDRESS IN OBSTETRICS.

By William Japp Sinclair, M.D., Professor of Obstetrics in the Owens College, Manchester.

In his address on obstetrics Professor Sinclair discussed the subject of Carcinoma in Women, chiefly in its Clinical Aspects. It is generally believed, he said, that carcinoma of the sexual organs of women is as cancer occurring in men, and to this extent therefore the subject may not appear to be one of obstetric medicine pure and simple. But malignant disease has a tendency to affect certain organs in women to an extent and with a relative frequency unknown in the other sex; and the clinical facts in the typical cases of cancer of the uterus make up a *tout ensemble*, an association of syndromata, a personality of the disease, that distinguish it from the malignant disease of any other organ. Then the frequency of its occurrence, the sex of its victims, and the pitiful character of its course from the social point of view lend it an interest and a pathos almost entirely its own.

### Scientific *versus* Clinical Work.

In discussing the nature of the disease Professor Sinclair pointed out the tendency which there had been to establish a sort of contrast—a certain antithesis—betwixt clinical and scientific work. Enlightened and patient industry in the observation and treatment of disease are, he said, the only sure way to professional distinction and honour, but now, since the modern development of pathology and bacteriology, the unknown or misunderstood is apt to be accepted as magnificent by the whole medical profession, and thus a certain distinction can be achieved without much talent or industry; the microtome and the cultivation tube—though work connected with them often resembles a sad mechanic exercise—have provided a royal road for many men into fields of clinical work which they have not known how to cultivate. They have shirked their apprenticeship to clinical medicine, yet claim the consideration and emoluments due to the skilled and experienced journeyman. Nevertheless one must hold in highest esteem and admiration the magnificent intellectual labours of the pioneers and masters of modern pathology and bacteriology.

### Sterile Histology.

After a prolonged account of the various views which have been taken from time to time by pathologists in regard to the nature and origin of the disease, Professor Sinclair comes to the somewhat depressing conclusion that we are not far from where we were at the beginning. We search in vain, he said, through the recent manuals of gynæcology and even of pathology and the records of pathological investigation, for any hint of progress in the evolution of an etiology of malignant disease of the internal sexual organs of women. Of histology there is enough and to spare, but of pathology in the narrower sense there is little to arrest attention, much less to satisfy; and there is absolutely nothing to indicate an advance from the position of a former generation of pathologists. The nomenclature is new—the thoughts and principles are old. There would, in fact, be a certain amount of truth in the allegation, were one to say that the diligent pursuit of the histology of malignant growths and other tumours had distracted the attention, the activities, and the energy, to say nothing of the intellectual force, which might have led to a true pathology of cancer of the uterus had attention been fixed upon structure, growth, and extension in relation to clinical phenomena. We have heard too much of cancer as neoplasm, too little of it as a disease.

If, now, we set aside all considerations of the etiology and pathology of cancer of the uterus and keep in mind only the clinical aspects of the disease, we see much ground for congratulation on the achievements of the last quarter of a century. The clinical work has been wholly surgical. What is not surgical is futile—it is hardly knowledge.

### Vaginal Hysterectomy.

Referring to vaginal hysterectomy, he said that whatever one may think of the remote results, no one who knows the history can deny that the reduction of the immediate mortality from this operation marks one of the greatest triumphs of modern surgery; for vaginal hysterectomy, when the disease is confined to the uterus, is not now more dangerous than an ordinary ovariotomy.

Yet in recent years a wave of depression has been passing over the gynæcological world on account of the unfavourable remote results of vaginal hysterectomy for cancer. At a recent congress of American gynæcologists one of the members said: "There are some men who claim for total extirpation of the uterus the happiest results. . . . Other men have looked upon the matter with utter hopelessness. . . . I am sorry to say that I have brought myself among the latter class from bitter experience." This, however, is far from being the view held by the majority of operators. So far, in fact, is this pessimistic view from being the true one, that the results obtained by many of the best-known operators continue to improve. They are obtaining better immediate results, and the number of "cures" increases—that is, the percentage of immunity for at least five years after operation becomes greater. The most remarkable figures of all are those given by Schuchardt as the results of the paravaginal operation. The best results before were those of Leopold in Dresden, who, with an operability of 20·4 per cent., had 50 per cent. of conventional "cures." Schuchardt raised the operability to 61 per cent., and had conventional cures to the extent of 40 per cent. In easy cases he had the astonishing result of 80 per cent. of cures.

### "Murderous Vivisections."

Meanwhile one of the results of the depression arising from the recognition of the unfavourable remote results of vaginal hysterectomy has been a rush, especially in Germany, to the extreme verge of the practical in dissecting out not only the internal sexual organs but the whole of the lymphatics and cellular tissue of the pelvis by the abdominal route. This has been declared to be justified by the belief that better remote results would be obtained than by vaginal hysterectomy.

From a careful and laborious attempt to keep up with the record of proceedings, I have no hesitation in saying, declares Professor Sinclair, that a large portion of the extended radical abdominal hysterectomies for cancer are murderous vivisections, which nothing hitherto advanced in their support appears to palliate, much less to justify. Most of the cases recorded have been too far advanced for any operation, however radical. "The radical nature of these operations," says one German critic, "is expressed only in the sad immediate and remote results, a high primary mortality, and injuries to the ureters and bladder in those who survive for a time." The operators profess to dissect out the pelvic, iliac, hypogastric, sacral, inguinal and lumbar lymphatic glands; and they resect the ureters and riemplant them in the bladder when they interfere with the scope of their proceedings.

The immediate mortality is terrific, but then, as Jacobs said, at Amsterdam, "the operation mortality is no longer an argument capable of arresting our efforts." And so far as experience of the remoter results has ripened, all admit that the patients who escape with their lives from the operations, reckoning only the possession of a permanent urinary fistula or the loss of a kidney among the consequences, are no better off in relation to recurrence than those who have undergone the comparatively safe operation of radical vaginal extirpation.

### The Direction of Future Progress.

Professor Sinclair holds then that the presently existing pessimism is not justified by the facts; that there is much reason for encouragement to greater efforts in the future; but that our hopes must rest on early vaginal operation, and not on the so-called extended abdominal sections. As far as the medical profession is concerned, the chief difficulty in the way of obtaining better results by early operation is that of early diagnosis. Anyone engaged in the practice of medicine should have no difficulty in diagnosing cancer of the uterus when a cavity has formed or when the patient is suffering from one of the hypertrophic forms of epithelioma of the cervix.

But there are cases in which there is real difficulty in establishing early diagnosis of uterine cancer. Now it is mere mockery, and the suggestion of utter impracticability to tell a busy medical practitioner to snip bits off the affected part and subject them to microscopic examination. And then we must keep in mind that it is easy to place too great reliance on mere microscopic diagnosis. It is, for example, impossible to diagnose between benign adenoma of the corpus uteri and the malignant form of the disease by means of the microscope alone; the distinction is purely clinical, notwithstanding the air of scientific accuracy which the masters of the microtome assume. How, then, can early diagnosis of carcinoma of the cervix be established clinically? The answer is by the friability of the tissue, which is pathognomonic in this particular phase of malignant growth. The presence of friable tissue in the cervix uteri indicates the existence of disease which is clinically malignant whatever the microscope may say.

For the amelioration of this terrible disease, then, our hopes rest on surgery, and that surgery may be enabled to do its best we must rely, not on the extension of operative procedures into the brilliance of vivisections, but on the united efforts of all concerned to bring the cases under surgical treatment at the earliest possible stage.

